# 4428 Harnessing Community Paramedicine for Transformative Fall Prevention Solutions

**DOI:** 10.1017/cts.2020.271

**Published:** 2020-07-29

**Authors:** Carmen Quatman, Melinda Gabriel, David Wisner, Mark Weade, Jennifer Garvin, Elizabeth Sheridan, Jessica Wiseman, Catherine Quatman-Yates

**Affiliations:** 1 The Ohio State University; 2 Westerville Fire Division; 3 Upper Arlington Fire Division

## Abstract

OBJECTIVES/GOALS: Healthcare costs for falls are expected to reach nearly 55 billion dollars annually in the US by 2020. Leveraging 911 calls as trigger events to activate fall prevention solutions could transform our ability to identify high-risk individuals and significantly improve fall prevention strategies globally. METHODS/STUDY POPULATION: An innovative pilot program entitled Community-centered Fall Intervention Team (Community FIT). Community FIT that leverages 911 calls, implementation science approaches, community partnerships, and collaboration among multiple healthcare disciplines including physical therapists, community paramedics, physicians, and social service coordinators was used to design and implement a community paramedicine fall intervention program. 911 call reports from February 2016 – August 2019 were analyzed using time series analyses to measure community level outcomes in fall-related calls and transports. RESULTS/ANTICIPATED RESULTS: 224 grab bars were installed free of charge to local residents (averaging approximately $125 per home for modifications).Over an 18-month period, time series analysis indicated an approximate demonstrated a consistent drop in the average fall-related 911 calls per month from 11.6 to 4.5 calls (a change of 61.21%) and a decrease of 58% in the transport rates to the hospital for fall-related 911 calls. 911 referrals to the community paramedicine program have also increased by 83%, demonstrating increased activation of fall prevention strategies with Community FIT. DISCUSSION/SIGNIFICANCE OF IMPACT: Collectively, these pilot study results provide preliminary support for individual and system level improvements in fall prevention by leveraging 911 calls to activate a community medicine fall prevention program. Future studies are needed to determine reach, long-term effectiveness, and sustainability of the program. CONFLICT OF INTEREST DESCRIPTION: Johnson & Johnson Hip Fracture Advisory Board (not related to project submission)

